# A quality improvement project to improve attendance to the physical health clinic at Southwark team for early psychosis

**DOI:** 10.1192/bjo.2021.524

**Published:** 2021-06-18

**Authors:** Christina Huggins, Joanna Cranshaw, Lucy Pauli, Beate Haege

**Affiliations:** South London and Maudsley NHS Foundation Trust

## Abstract

**Aims:**

The aim of this project was to improve the booking and attendance of patients under Southwark Team for Early Psychosis (STEP) into the physical health clinic.

**Background:**

STEP is an Early Intervention Service which provides treatment to 230 adults (18-65 years) with first episode psychosis in the community. In line with national and trust guidance, physical health checks are completed at baseline, 3 months, 6 months and annually, through a weekly physical health clinic run by the core trainee (CT). This is an essential opportunity to assess and monitor patients’ physical health and aid decisions regarding psychotropic medications, which is particularly important given the increased morbidity and mortality in this group and their reduced engagement with health services. It was noted that attendance to the clinic was poor and there was no guidance about how to communicate the results to the General Practitioner (GP).

**Method:**

Data on the number of clinic appointments booked and attended were collected over 3 defined 9 week intervals between 17/09/18 and 29/07/19. The interventions were implemented prior to the third round of data collection and included an educational session to the STEP team and a protocol for booking and running the clinic to be used by the CT. We devised a physical health questionnaire to be completed by patients on arrival, which includes a summary of the Maudsley guidelines for antipsychotic monitoring. Finally, we created a template letter to communicate the results to the GP.

**Result:**

Following the interventions, the percentage of available clinic slots booked increased from an average of 27.8% to 100%. The proportion of slots attended reduced from an average of 80% to 50%. However, the absolute number of patients booked into clinic increased from an average of 10 patients over 9 weeks pre-intervention, of which an average of 8 patients attended, to 36 patients post-intervention, of which 18 attended.

**Conclusion:**

We observed full utilisation of available clinic slots post-intervention and an increase in the absolute number of patients attending. Given the ongoing use of the protocols developed, we expect these changes to be sustainable. The number of patients attending could be further increased by training additional staff to run the clinic more often and more flexibly. The number of Did-Not-Attends could be reduced by care-coordinators sending reminder texts prior to the appointments.

